# Gluteus medius muscle function in people with and without low back pain: a systematic review

**DOI:** 10.1186/s12891-019-2833-4

**Published:** 2019-10-22

**Authors:** Sean Sadler, Samuel Cassidy, Benjamin Peterson, Martin Spink, Vivienne Chuter

**Affiliations:** 1 Discipline of Podiatry, University of Newcastle, Ourimbah, NSW 2258 Australia; 2 Priority Research Centre for Physical Activity and Nutrition, University of Newcastle, Newcastle, Australia

**Keywords:** Low back pain, Systematic review, Gluteus medius, Electromyography

## Abstract

**Introduction:**

Globally, low back pain (LBP) is one of the greatest causes of disability. In people with LBP, dysfunction of muscles such as the gluteus medius have been demonstrated to increase spinal loading and reduce spinal stability. Differences in gluteus medius function have been reported in those with LBP compared to those without, although this has only been reported in individual studies. The aim of this systematic review was to determine if adults with a history, or current LBP, demonstrate differences in measures of gluteus medius function when compared to adults without LBP.

**Methods:**

MEDLINE, EMBASE, AMED, PsycINFO, PubMED, Pro Quest Database, CINAHL and SPORTDiscus were searched from inception until December 2018 for published journal articles and conference abstracts. No language restrictions were applied. Only case-control studies with participants 18 years and over were included. Participants could have had any type and duration of LBP. Studies could have assessed gluteus medius function with any quantifiable clinical assessment or measurement tool, with the participant non-weight bearing or weight bearing, and during static or dynamic activity. Quality appraisal and data extraction were independently performed by two authors.

**Results:**

The 24 included articles involved 1088 participants with LBP and 998 without LBP. The gluteus medius muscle in participants with LBP tended to demonstrate reduced strength and more trigger points compared to the gluteus medius muscle of those without LBP. The level of activity, fatigability, time to activate, time to peak activation, cross sectional area, and muscle thickness showed unclear results. Meta-analysis was not performed due to the heterogeneity of included studies.

**Conclusion:**

Clinically, the findings from this systematic review should be considered when assessing and managing patients with LBP. Future studies that clearly define the type and duration of LBP, and prospectively assess gluteus medius muscle function in those with and without LBP are needed.

**Trial registration:**

PROSPERO (CRD42017076773).

## Introduction

Low back pain (LBP) has been identified as the leading contributor of disability and was ranked sixth largest contributor to the burden of global disease, costing individuals and governments billions of dollars in both direct and indirect costs annually [1]. The prevalence of LBP increases linearly after the third decade of life [2], and, with an ageing population, the prevalence and impact of this condition are expected to increase [1].

Dysfunction of muscles of the lumbopelvic-hip complex is a hallmark of LBP [3]. At the hip, individuals with LBP are more likely to exhibit reduced gluteus medius muscle strength [4], reduced hip abduction force output [5], and altered hip muscle recruitment, demonstrating a distal-to-proximal muscle activation pattern in the lower limb compared to proximal-to-distal in healthy controls [6]. These alterations to gluteus medius muscle function and strength have been suggested to lead to LBP [7], however, it is unknown whether such muscle deconditioning or atrophy is the cause or result of symptomatic LBP.

The gluteus medius is one of the main pelvic stabiliser muscles and plays a significant role in controlling transverse and frontal plane motion of the femur and hip [8], providing stability to the lumbopelvic-hip complex [9]. This stability may be important in controlling excessive movement and allowing adequate attenuation of forces throughout the lower back region. Gluteus medius weakness and consequential loss of dynamic lateral stability of the pelvis and lower back is suggested to lead to increased lateral trunk flexion and subsequent intervertebral disc compression [10], as well as altered movement patterns which may contribute to the development or exacerbation of LBP during standing [11–15].

Individual studies have found differences in the activation, strength, and number of trigger points in the gluteus medius muscle between those with and without LBP [12, 16–18]. Due to these differences in gluteus medius muscle function, perhaps this muscle has a role in either the development or exacerbation of LBP. The mechanism by which this occurs is suggested to relate to the role in which the gluteus medius muscle plays in providing both frontal and transverse plane stability of the pelvis and lower back [13–15]. Determining the nature of gluteus medius function in those with LBP compared to those without is a key component to more effective assessment techniques and management of the condition. Therefore, a systematic review that collectively evaluates gluteus medius function in those with and without LBP is required.

This systematic review aims to determine, by review of case-control studies, if adults with a history of, or current LBP, demonstrate differences in measures of gluteus medius function when compared to adults without LBP. A secondary aim is to investigate if there is a difference in gluteus medius muscle function between types and durations of LBP.

## Methods

### Search strategy

This systematic review was registered with PROSPERO (CRD42017076773) and has been reported in accordance with the PRISMA statement [19]. MEDLINE, EMBASE, AMED, PsycINFO, PubMED, Pro Quest Database, CINAHL and SPORTDiscus were searched from inception until 14th December, 2018. No language restrictions were applied to published articles or conference abstracts. Keywords were truncated and combined using AND/OR, with search terms adapted for each of the databases (Additional file 1).

### Eligibility criteria

Case-control studies including participants 18 years and older with LBP of any type (specific or non-specific) and of any duration (acute, subacute or chronic) were eligible for inclusion. Studies measuring gluteus medius function in any way, for example, strength, flexibility, fatigability, percentage of maximum voluntary contraction, cross-sectional area, timing or extent of contraction, or other unidentified measurement were eligible for inclusion. Studies could assess gluteus medius with any quantifiable clinical assessment or measurement tool, with the participant non-weight bearing or weight bearing, and during static or dynamic activity.

Studies were excluded if they included participants that were pregnant, had a history of low back surgery, or were solely investigating the effect of an intervention on the gluteus medius muscle.

### Study selection

One reviewer conducted the electronic searches (SS). Two reviewers (SC/SS) independently screened citations at title and abstract level. One reviewer (SS) retrieved potentially eligible full text articles and these were assessed independently by two reviewers (SC and SS). Authors were contacted where clarification was required for assessing eligibility for inclusion. There were no disagreements so there was no need to seek arbitration by a third reviewer (VC). Data were independently extracted by two reviewers (SC and BP), using a standardised data extraction form, and cross checked by a third reviewer (SS). For the purposes of study classification we defined duration of back pain as: Acute (< 6 weeks), subacute (6- < 12 weeks), and chronic (≥12 weeks) [20]. Due to the heterogeneity between studies, a meta-analysis was not performed.

### Quality assessment

Two reviewers (SC/BP) independently appraised eligible full text articles using the CASP tool for case-control studies. The results of quality appraisal were checked by a third reviewer (SS) and no disagreements occurred.

## Results

### Study identification

**Fig. 1:**
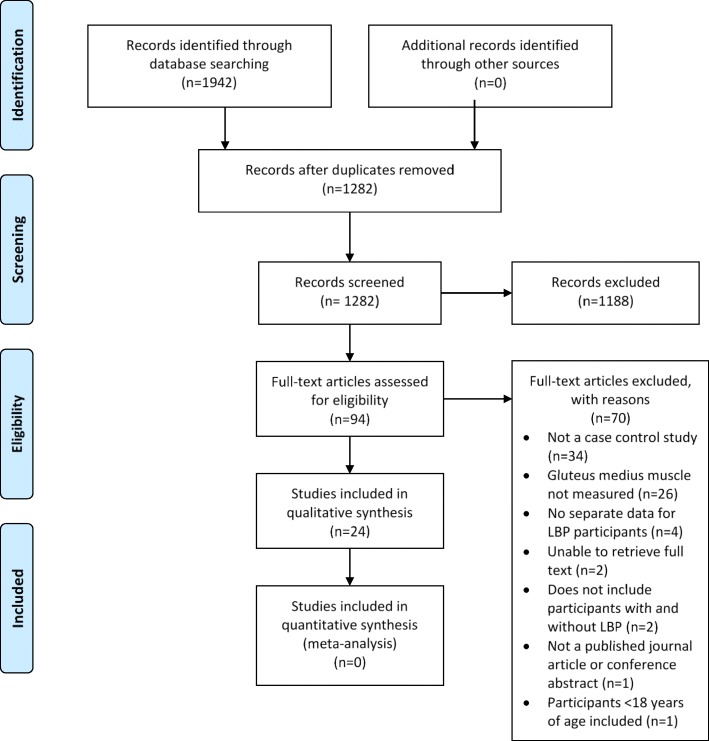
PRIMSA flow diagram

Searches retrieved 1942 citations of which 94 were eligible for full text review. After review, 24 full text articles of mixed methodological quality (Additional file 2) were included, while 70 were excluded (Additional file 3) based on exclusion criteria (Fig. 1).

After contacting authors for eligibility confirmation, one conference abstract was excluded as it was confirmed it duplicated published journal article data [5]. Two full text articles could not be retrieved and were not provided by authors [21, 22] so were subsequently excluded.

### Characteristics of included studies

The 24 articles [3–6, 16–18, 23–39] investigating gluteus medius function included 1088 participants with LBP and 998 healthy controls without LBP (Table 1). Studies included one or more of the following gluteus medius measurement outcomes: electromyographic (EMG) activity level [17, 18, 26, 28, 29, 31, 34, 38], EMG fatigability [24, 31, 34], EMG time to onset [6, 26, 29, 30], EMG time of peak activation [6, 17, 18, 24, 26, 28–31, 34, 38], hip abductor strength [3–5, 16, 25, 29], Trendelenburg sign [5, 16] hip abductor torque [23, 33], trigger points [16, 27, 35, 36], cross sectional area [32, 37], or muscle thickness [39]. Studies conducted these measurements non-weight bearing [3, 4, 6, 25, 27, 30, 32–37, 39], dynamically weight bearing [17, 23, 26, 38] or statically weight bearing [24, 28, 31]. Three studies utilised non-weight bearing measurements while also using dynamic and static measurements while weight bearing [5, 16, 29].

**Table 1:** Overview of included studies

Study	Participants LBP Group	Participants control group	Type of LBP	Duration of LBP	Presence of LBP at Baseline	Gluteus medius measurement outcome
Aboufazeli et al. 2018 [39] Iran.	*n* = 30 Mean age, years (SD): 34.6 (6.2) Population: Not reported Gender: 100%F BMI (SD): 23.4 (3.2)	*n* = 30 Mean age, years (SD): 36.7 (6.7) Population: Note reported Gender: 100%F BMI (SD): 23.6 (3.3)	Nonspecific LBP	≥3 months	Yes	Muscle thickness
Arab & Nourbakhsh 2010 [4] Iran	*n* = 100 Mean age, years (SD): 42.58 (14.1) Population: Patients of orthopaedic department Gender: Not Reported BMI (SD): 25.03 (3.0)	*n* = 100 Mean age, years (SD): 43.40 (4.41) Population: Patients of orthopaedic department Gender: Not Reported BMI (SD): 25.68 (4.1)	Not Reported	> 6 weeks	Yes	Strength
Cai & Kong 2015 [23] Singapore	*n* = 18 Mean age, years (SD): 27.80 (NR) Population: Recreational Runners Gender: 50%F BMI (SD): 21.75 (NR)	*n* = 18 Mean age, years (SD): 24.60 (NR) Population: Recreational Runners Gender: 50%F BMI (SD): 21.40 (NR)	Not Reported	> 3 months, but less than 36 months	Yes	Strength
Cooper et al. 2016 [16] USA	*n* = 150 Mean age, years (SD): 41.40 (13.0) Population: Patients at Iowa Spine Centre Physical Therapy Clinic Gender: 64.70%F BMI (SD): 29.60 (7.2)	*n* = 75 Mean age, years (SD): 40.70 (13.9) Population: Patients at Iowa Spine Centre Physical Therapy Clinic Gender: 64.30%F BMI (SD): 25.80 (7.0)	Nonspecific LBP	> 3 months	Yes – Only one participant reported no current LBP	Strength and trigger points
Embaby & Abdallah 2013 [24] Egypt	*n* = 15 Mean age, years (SD): 29.53 (2.4) Population Clinical Instructors Gender: 100%F BMI (SD): 24.01 (NR)	*n* = 15 Mean age, years (SD): 29.07 (2.4) Population: Clinical Instructors Gender: 100%F BMI (SD): 22.63 (NR)	Nonspecific	Periods of mild or moderate LBP for > 6 months	Not Reported	Fatigability
Farahpour et al. 2018 [17] Iran	*n* = 15 Mean age, years (SD): 25.30 (2.9) Population: Patients from a ‘clinic’ Gender:0%F BMI (SD): 26.80 (1.5)	*n* = 15 Mean age, years (SD): 26.00 (2.9) Population: Not reported Gender: 0%F BMI (SD): 25.90 (3.2)	Not reported	Not reported	Yes	Activity level
Farasyn & Meeusen 2005 [35] Belgium	*n* = 87 Mean age, years (SD): 43.00 (13.0) Population: Physiotherapy patients Gender: 55.17%F BMI (SD): 20.50 (2.8)	*n* = 64 Mean age, years (SD): 40.00 (11.0) Population: Physiotherapy patients Gender: 62.50%F BMI (SD): 21.50 (3.2)	Nonspecific LBP	Subacute nonspecific lower back pain	Yes	Trigger points
Hides et al. 2016 [25] Australia	As a whole group LBP *n* = 7, no LBP *n* = 18 Mean age, years (SD): 24.40 (5.5) Population: Elite Footballers Gender: 0%F BMI (SD): 23.61 (NR)		Not Reported	Not reported	Not reported	Strength
Hungerford et al. 2003 [26] Australia	*n *= 14 Mean age, years (SD): 32.70 (NR) Population: Men with SIJP Gender: 0%F BMI (SD): 24.63 (NR)	*n* = 14 Mean age, years (SD): 33.50 (NR) Population: without SIJP Gender: 0%F BMI (SD):23.40 (NR)	SIJP	> 2 months	Yes	Activity level and time to onset
Iglesias-Gonzalez et al. 2013 [27] Spain	*n* = 42 Mean age, years (SD): 45.00 (10.0) Population: Patients of a private physical therapy clinic Gender: 50.00%F BMI (SD):24.50 (3.2)	*n* = 42 Mean age, years (SD): 45.00 (9.0) Population: Subjects who responded to local advertisements Gender: 50.00%F BMI (SD): 24.90 (3.4)	Nonspecific LBP	> 3 years	Yes	Trigger points
Kendall et al. 2010 [5] Canada	*n* = 10 Mean age, years (SD): 32.00 (NR) Population: Not Stated Gender: 80.00%F BMI (SD): 20.86 (NR)	*n* = 10 Mean age, years (SD): 26.00 (NR) Population: Not Stated Gender: 80.00%F BMI (SD): 21.61 (NR)	Nonspecific LBP	> 6 weeks	Yes	Strength
Larsen et al. 2018 [38] Denmark	*n* = 27 Mean age, years (SD): 27.40 (9.9) Population: University campus and hospital Gender: 44.44 BMI (SD): 21.90 (3.2)	*n* = 26 Mean age, years (SD): 23.60 (4.4) Population: University campus and hospital Gender: 61.53%F BMI (SD): 23.80 (2.5)	Nonspecific	> 3 years	No	Activity level
Mendis et al. 2016 [37] Australia	As a whole group LBP *n* = 13, no LBP *n* = 33 Mean age, years (SD): 22.80 (3.5) Population: Australian Elite AFL Players Gender: 0%F BMI (SD): 25.00 (NR)		Not Reported	Not Reported	Yes	Cross-sectional area
Nelson-Wong et al. 2013 [6] USA	*n* = 17 Mean age, years (SD): 27.71 (10.6) Population: general population Gender: not reported BMI (SD): 23.42 (2.9)	*n* = 17 Mean age, years (SD): 28.52 (10.2) Population: general population Gender: not reported BMI (SD): 22.99 (1.8)	Not Reported	Not Reported	Yes	Time to onset
Njoo & Van der Does 1994 [36] Netherlands	*n* = 61 Mean age, years (SD): 36.20 (9.8) Population: Patients of participating health care centres Gender: 44.20%F BMI (SD): Not Reported	*n* = 61 Mean age, years (SD): 38.10 (9.9) Population: Every 10th patients of health care centres without LBP Gender: 50.70%F BMI (SD): Not Reported	Nonspecific LBP	Recent episode of less than 2 months	No	Trigger points
Notzel et al. 2011 [28] Germany	*n* = 8 Mean age, years (SD): 42.40 (14.5) Population: Not Reported Gender: 100%F BMI (SD): 23.10 (2.4)	*n* = 12 Mean age, years (SD): 27.30 (7.1) Population: Not Reported Gender: 100%F BMI (SD): 20.40 (2.6)	Nonspecific LBP	> 6 months several times per week or daily	Not Reported	Activity level
Nourbakhsh & Arab 2002 [3] Iran	*n* = 300 Mean age, years (SD): 43.00 (NR) Population: Hospital inpatients Gender: 50.00%F BMI (SD): 25.76 (NR)	*n* = 300 Mean age, years (SD): 43.00 (NR) Population: Hospital inpatients Gender: 50.00%F BMI (SD): 24.44 (NR)	Not Reported	> 6 weeks	Yes	Strength
Penney et al. 2014 [29] Canada	*n* = 21 Mean age, years (SD): 46.00 (15.2) Population: Patients at local physiotherapy clinics Gender: 42.85%F BMI (SD): 27.40 (NR)	*n* = 22 Mean age, years (SD): 44.00 (15.5) Population: University and Hospital Community Gender: 33.36%F BMI (SD): 26.95 (NR)	Nonspecific LBP	> 12 weeks	Yes	Activity level, time to onset, and strength
Rabel et al. 2013 [30] USA	*n* = 12 Mean age, years (SD): 44.40 (14.6) Population: Physiotherapy patients Gender: 58.33%F BMI (SD): 29.70 (10.3)	*n* = 22 Mean age, years (SD): 27.20 (4.6) Population: Recreationally active Gender: 50.00%F BMI (SD): 24.70 (4.9)	Not Reported	< 2 months and > 3/10 on VAS	Yes	Time to onset
Ringheim et al. 2015 [31] Norway	*n* = 17 Mean age, years (SD):39.00 (5.4) Population: Hospital outpatients Gender: 58.82%F BMI (SD): 25.90 (4.7)	*n* = 20 Mean age, years (SD): 40.20 (5.4) Population: Not Reported Gender: 61.90%F BMI (SD): 25.20 (3.7)	Nonspecific	> 3 months	Yes	Activity level and fatigability
Santos et al. 2013 [18] Brazil	*n* = 29 Mean age, years (SD): 45.80 (14.3) Population: Orthopaedic patients Gender: 100%F BMI (SD): 24.15 (3.9)	*n* = 30 Mean age, years (SD): 44.57 (13.6) Population: Not Reported Gender: 100%F BMI (SD): 23.77 (2.1)	Nonspecific LBP	> 3 months	No	Activity level and time of peak
Skorupska et al. 2016 [32] Poland	*n* = 71 Mean age, years (SD): 47.70 (8.4) Population: Not reported Gender: 61.97%F BMI (SD): Not Reported	*n* = 29 Mean age, years (SD): 47.60 (9.9) Population: Not reported Gender: 65.51%F BMI (SD): Not Reported	Not Reported	Subacute or Chronic	Yes	Cross-sectional area
Sutherlin & Hart 2015a [33] USA	*n* = 12 Mean age, years (SD): 24.00 (4.0) Population: Not Reported Gender: Not Reported %F BMI (SD): 25.19 (3.4)	*n* = 12 Mean age, years (SD): 22.00 (3.0) Population: Not Reported Gender: Not Reported %F BMI (SD): 21.28 (2.4)	Not Reported	Not reported	Yes	Strength
Sutherlin & Hart 2015b [34] USA	*n* = 12 Mean age, years (SD): 24.00 (4.0) Population: Not Reported Gender: 58.33%F BMI (SD): 25.19 (3.4)	*n* = 12 Mean age, years (SD): 22.0 (3.0) Population: Not Reported Gender: 75.00%F BMI (SD): 21.28 (2.4)	Not Reported	Not reported	Yes	Activity level and fatigability

*SD* standard deviation, *BMI* body mass index, *LBP* low back pain, *NR* not reported, *SLR* single leg raise, *T12* 12th thoracic vertebra, *SIJP* Sacroiliac joint pain, *SIJ* Sacroiliac joint

All studies included participants with either nonspecific or unidentified LBP (Table 1). The length of time participants had LBP within each of the studies varied. Some included acute [25], subacute [4, 35], chronic [16, 18, 23, 24, 27–29, 31, 38, 39], or mixed/unclear durations of LBP [3, 5, 6, 17, 26, 30, 32–34, 36, 37].

Most studies performed generally well on the quality appraisal tool (Additional file 2). However, in the majority of studies there was insufficient information to determine how controls were recruited and there were inconsistencies in the type and number of potential confounding factors that were addressed.

### Included studies by measurement outcome

#### Level of muscle activity (EMG)

Eight studies [17, 18, 26, 28, 29, 31, 34, 38] measured the amount of activity in the gluteus medius muscle with EMG (Table 2). Of these studies, five [18, 26, 28, 29, 31] measured gluteus medius muscle activity in a static weight bearing position, with mixed results. Two studies found less gluteus medius muscle activity in those with LBP [18, 28], one study found more activity in those with LBP [29], and the remaining two studies found no difference in gluteus medius muscle activity between those with and without LBP [26, 31]. A further two studies measured gluteus medius muscle activity dynamically, with one study finding less gluteus medius muscle activity in those with LBP [17], and the other study finding no difference between those with and without LBP [38]. The remaining study measured gluteus medius muscle activity in a non-weight bearing position and found no difference in gluteus medius muscle activity between those with and without LBP [34].

**Table 2:** Included studies per outcome measurement

Study	Measurement equipment	Method	Result				Major Conclusions
Notzel et al. 2011 [28]	Modified Posturomed, Biovision	Participants stood barefoot in a static weight bearing position on the posturomed plate for 10 s while it that vibrated.	Activity level	Fatigability	Time to onset	Time of peak	Patients with LBP demonstrated statistically significant less gluteus medius muscle activity compared to controls. This could be associated with reduced hip stability.
			LBP: 56.29 μV (±39.63) nLBP: 96.42 μV (±64.77), *p* < .05 Normalisation: not performed, raw EMG values used	Not measured	Not measured	Not measured	
Farahpour et al. 2018 [17]	BTS FREE EMG300	Participants walked for 8 steps in standardised shoes.	RMS LBP: 111.8% (±48.6%) nLBP: 48.4% (±27.3%), *p < .05* Normalisation: % of MVIC in single leg stance while maintaining the pelvis level	Not measured	Not measured	Not measured	Participants with LBP showed statistically significant more gluteus medius muscle activity compared to controls*.*
Larsen et al. 2018 [38]	Noraxon EMG	Participants performed 10 ascent and 10 descent step tasks at self-selected speed, separated by 3 min of rest.	RMS Specific values not reported, *p* > .05 Normalisation: % of sub maximum voluntary contraction during standing hip abduction with manual external resistance	Not measured	Not measured	Not measured	No statistically significant differences in gluteus medius activity during ascent and descent between those with and without LBP.
Penney et al. 2014 [29]	Biopac EMG system	Participants stood in a single leg stance position for 30 s, with the non-weight bearing limb flexed between 60 and 90 degrees at the hip. One minute rest between each of the 3 reps per side, with a 5 min rest between sides.	RMS LBP: 5.8% (± 2.6%) nLBP: 4.2% (± 2.3%), *p* = .05 iEMG LBP: 122% (± 55%) nLBP: 87.8% (± 49%), *p* = .03 Normalisation: % of MVIC in side-lying hip abduction with manual resistance	Not measured	LBP: 461.7 ms (±286.5) nLBP: 493.4 ms (±292.8), *p* = .73	Not measured	There was no statistically significant difference in onset time of the gluteus medius when moving to unipedal stance between the groups. However, the LBP group demonstrated statistically significant more gluteus medius activation.
Santos et al. 2013 [18]	EMG810C, EMG System do Brasil®	Participants started kneeling and then were asked to flex their R hip and extend their R knee until the R foot contacted the ground (the L knee remained on the ground). The same process was then repeated on the L limb.	R peak amplitude, MED (IQR_1–3_) LBP: 1.25 (1.00–2.16) nLBP: 1.60 (1.00–2.10), *p* = .007 L peak amplitude, MED (IQR_1–3_) LBP: 1.19 (1.04–2.31) nLBP: 1.81 (1.02–2.11), *p* < .001 R iEMG, MED (IQR_1–3_) LBP: 0.66 (0.17–1.00) nLBP 1.00 (0.35–1.48), *p* = .004 L iEMG, MED (IQR_1–3_) LBP: 1.00 (0.57–1.00) nLBP 1.00 (0.87–2.00), *p* = .001 Normalisation: % of average activity during the kneeling task.	Not measured	Not measured	R, % of duration of task, MED (IQR_1–3_) LBP: 0.68 (0.11–0.94) nLBP: 0.44 (0.07–0.74), *p* = .001 L, % of duration of task, MED (IQR_1–3_) LBP: 0.86 (0.13–1.00) nLBP: 0.21 (0.05–0.83), *p* < .001	Participants with LBP demonstrated statistically significant lower amounts of glutues medius muscle activity, and later times of peak activation compared to those without LBP.
Ringheim et al. 2015 [31]	EMG TeleMyo 2400 (Noraxon)	Participants stood barefoot for 15 min.	Start RMS (%Max) LBP: 10.4 (6.3–36.5) nLBP 8.3 (4.9–11.6), *p =* .19 Slope RMS (%Max) LBP: − 1.5 (− 9.1–7.7) nLBP: − 0.6 (− 1.7–3.5), *p* = .66 Normalisation: % of maximum voluntary contraction during standing hip flexion and extension in an isokinetic device.	Coefficient of variation LBP: 27.4 (23.4–48.5) nLBP: 31 (17.5–39.7), *p =* .62 Slope MDF (Hz/min) LBP: 12.9 (− 9.0–21.3) nLBP: 2.5 (− 8.0–21.3), *p* = .28	Not measured	Not measured	No statistically significant differences in the amount of gluteus medius muscle activity or variability of muscle activity over time between those with and without LBP.
Embaby et al. 2013 [24]	Myomonitor® Wireless EMG System	Participant stood shod for 30 min.	Not measured	R first 5 min, MDF LBP: 172.40 (±48.96) nLBP: 171.41 (±38.87), *p* > .05 R Last 5 min, MDF LBP: 158.91 (±49.03) nLBP: 195.19 (±34.74), *p* < .05 L First 5 min, MDF LBP: 159.29 (±48.81) nLBP: 173.12 (±41.36), *p* > .05 L Last 5 min, MDF LBP: 177.18 (±53.95) nLBP: 185.04 (±48.04), *p* > .05	Not measured	Not measured	Participants with LBP demonstrated statistically significant less gluteus medius muscle activity on the R during the last 5 min compared to those without LBP (indicating greater fatigue). Differences in the first 5 min on both sides, and the last 5 min on the L side, were not statistically significant.
Hungerfor-d et al. 2003 [26]	Noraxon Telemyo 8 EMG	Participants stood on one leg then flexed the contralateral hip and knee to 90 degrees. Five trials per side were conducted.	Peak amplitude Specific values for gluteus medius not reported, *p* > .05 Normalisation: % of maximal activity during the single leg standing task.	Not measured	Specific values (in ms) for gluteus medius not reported, *p* > .05	Not measured	No statistically significant differences in the amount of activity or time of onset of the gluteus medius muscle in those with and without LBP.
Sutherin et al. 2015b [34]	EMG100C Biopac	Participants performed 5 consecutive isometric hip abduction contractions, in a side-lying position at zero degrees of hip abduction, each lasting 30 s. This was done on both sides, separated by 15 min of rest.	RMS No specific values reported, *p* > .05 Normalisation: % of MVIC during side-lying hip abduction with manual resistance.	MDF No specific values reported, *p* > .05	Not measured	Not measured	No statistically significant differences in the amount or duration of gluteus medius muscle activity between those with and without LBP.
Nelson-Wong et al. 2013 [6]	Biopac MP150	Participants performed the active hip abduction (AHAbd) test in a side-lying position. Note: A positive value indicates the first listed muscle activates first and a negative value indicates the second listed muscle activates first.	Not measured	Not measured	REO-RGMd LBP: − 0.18 s (±0.28) nLBP: 0.10s (±0.31), *p* = .015 LEO-RGMd LBP: − 0.03 s (±0.37) nLBP: 0.03 s (±0.37), *p* = .65 RIO-RGMd LBP: − 0.11 s (±0.33) nLBP: 0.14 s (±0.33), *p* = .033 LIO-RGMd LBP: 0.02 s (±0.37) nLBP: 0.08 s (±0.40), *p* = .62 RES-RGMd LBP: 0.05 s (±0.34) nLBP: 0.06 s (±0.33), *p* = .94 LES-RGMd LBP: − 0.11 s (±0.29) nLBP: 0.07 s (±0.36), *p* = .15 REO-LGMd LBP: 0.17 s (±0.38) nLBP: 0.05 s (±0.30), *p* = .35 LEO-LGMd LBP: 0.04 s (±0.39) nLBP: 0.12 s (±0.35), *p* = .55 RIO-LGMd LBP 0.01 s (±0.42) nLBP: 0.19 s (±0.28), *p* = .15 LIO-LGMd LBP: − 0.04 s (±0.32) nLBP: 0.17 s (±0.35), *p* = .049 RES-LGMd LBP: − 0.24 s (±0.33) nLBP: 0.09 s (±0.39), *p* = .014 LES-LGMd LBP: 0.01 s (±0.34) nLBP 0.10 s (±0.38), *p* = .44	Not measured	During the R AHAbd test, participants with LBP demonstrated statistically significant earlier activation of the R gluteus medius muscle relative to the ipsilateral trunk flexors (RIO and REO), compared to controls. During the L AHAbd test, participants with LBP statistically significantly activated the LGMd prior to the contralateral trunk extensors (RES) and ipsilateral IO, compared to controls.
Rabel et al. 2013 [30]	Noraxon Telemyo 2400 T EMG	Participants performed the active hip abduction (AHAbd) test in a side-lying position. Note: the larger the number, the longer it took for that muscle to activate.	Not measured	Not measured	LBP: 1629 ms (±1715) nLBP: 648 ms (±150), *p* = .115	Not measured	No statistically significant differences in time to onset for the gluteus medius muscle in those with and without LBP.
Hides et al. 2016 [25]	Power Trak II handheld dynamometer	Participants were positioned supine with hip in neutral. A strap was used to stabilise pelvis. Participants abducted their hip against the dynamometer at a maximal effort for 5 s with examiner resistance applied. Three trials with a 15 s rest between each trial.	Strength				Those with LBP had statistically significantly less gluteus medius muscle strength on the stance limb, but significantly more on the kicking limb.
			Stance leg LBP: 154.1 Nm (±10.0) nLBP: 161.5 Nm (±6.6), *p* < .05 Kicking Leg LBP: 165.1 Nm (±11.8) nLBP: 143.9 Nm(±7.8) *p* < .05				
Kendall et al. 2010 [5]	Lafayette manual muscle tester	The test limb was positioned parallel to the treatment table, directly in line with the hip. 3 maximal voluntary isometric strength contractions with a 30s rest period between trials was performed	LBP: 6.6 (N/kg) (5.4 to 7.7) nLBP: 9.5 (N/kg) (7.2 to 11.9) *p* = .03				LBP participants had statistically significantly less gluteus medius muscle strength compared to those without LBP.
Arab et al. 2010 [4]	Pressure meter	Side lying hip abduction test. Three maximal voluntary isometric contractions, held for 5 s with 15 s rest between trials.	LBP: 27.87 kPa (± 7.95) nLBP: 33.51 kPa (± 7.29), *p* < .001				LBP participants had statistically significantly less gluteus medius muscle strength compared with subjects without LBP.
Cai et al. 2015 [23]	Isokinetic dynamometer	Three standing concentric muscle contractions (torque) measured with leg secured to dynamometer	Male LBP: 1.49 (Nm/kg) (±0.39) Male nLBP: 1.52 (Nm/kg) (±0.41) Female LBP 1.05 (Nm/kg) (±0.39) Female nLBP: 1.17 (Nm/kg) (±0.35) *p* = .596 (Gp) *p* = .743 (Gp by Sex)				No statistically significant differences in gluteus medius strength between those with and without LBP.
Penney et al. 2014 [29]	Lafayette Manual Muscle Tester	Participants were laid on their side and abducted their hip whilst the examiner resisted with their hand just superior to the ankle. Two maximal resisted voluntary contractions for a 3s max voluntary contraction with 1 min rest in-between.	Right LBP: 1.04 (N/Kg) (± 0.32) Right NLBP: 1.36 (N/Kg) (±0.33) Left LBP: 1.05 (N/Kg) (± 0.26) Left nLBP: 1.23 (N/Kg) (±0.30) *p =* .04 (right) *p* = .002 (left)				LBP participants had statistically significantly less gluteus medius muscle strength on both sides compared to those without LBP.
Nourbakh-sh et al. 2002 [3]	Pressure meter	Side lying hip abduction test. Three maximal voluntary isometric contractions, held for 5 s	LBP: 26 kPa (±8) nLBP: 32 kPa (±7), *p* < .01				LBP participants had statistically significantly less gluteus medius muscle strength compared to those without LBP.
Sutherlin et al. 2015a [33]	Isokinetic dynamometer	Side lying hip abduction, three maximal voluntary isometric contraction (torque). Hip-abduction trials lasting 5 s were recorded, with 30 s of rest between trials.	LBP: 1.64 (Nm/Kg) (±0.44) nLBP: 1.65 (Nm/Kg) (± 0.28), *p* = .944				No statistically significant differences in gluteus medius strength between those with and without LBP.
Cooper et al. 2016 [16]	Subjective Measure	Gluteus medius strength was tested by placing subject in side-lying and having the subject abduct and slightly extend the hip while keeping the pelvis rotated slightly forward. Resistance was applied at the ankle. Graded 1–5.	LBP: 3.35 (±0.73) nLBP: 4.46 (±0.50), *p* < .001				LBP participants had statistically significantly less gluteus medius muscle strength compared to those without LBP.
Cooper et al. 2016 [16]	Subjective observation	While standing one hip is flexed. Trendelenburg sign considered present if the subject was unable to maintain the pelvis level or had to shift the trunk to keep the pelvis level.	Presence of Trendelenburg sign occurred 54.2% of the time in those with LBP compared to 9.7% of the time for those in the no LBP group *p* < .001				LBP participants were statistically significantly more likely to demonstrate a trendelenburg sign, indicating gluteus medius muscle weakness.
Kendall et al. 2010 [5]	Treadmill and Vicon	Subjects performed a baseline standing trial, 2 static Trendelenburg trials, and a 30s walking trial on a treadmill at a speed of 1.34 m/s.	LBP right: − 1.9 deg (− 7.0 to 1.7) nLBP right: − 2 deg (− 4.8 to 1.2) LBP left: − 1.6 deg (− 1.6 to 2.6) nLBP left: − 2.2 deg (− 4.3 to 0.7) Negative values indicate hip hike; positive values indicate pelvic drop. No significant differences.				No statistically significant differences in presence of Trendelenburg sign between those with and without LBP, indicating no difference in dynamic strength provided by the gluteus medius.
Farasyn et al. 2005 [35]	Fischer pressure algometer	Lying prone the rate of pressure increase was maintained at a constant rate of on average 1Kg/sec. Three short consecutive PPT measurements with 10 s in between were performed.	Trigger points				Participants with LBP had a statistically significant lower threshold for pain than those without LBP.
			LBP: 6.1 kg/cm^2^ (±1.6) nLBP: 7.2 kg/cm^2^ (± 1.5), *p* < .001				
Cooper et al. 2016 [16]	Palpation	Gluteus medius was palpated from its insertion, muscle belly and origin. Tenderness was defined as pain reported by patient and when using enough pressure to blanch the examiner’s nail.	LBP (affected side): Tenderness associated with triggers points was more prevalent (68.10%) on the side of the body affected by LBP nLBP: Tenderness associated with triggers points occurred in 11.20% of the gluteus medius muscles of those without LBP *p* < .001 LBP (affected side): Tenderness associated with triggers points was more prevalent (68.10%) on the side of the body affected by LBP LBP (unaffected side): Tenderness associated with trigger points was less prevalent (4.80%) on the side of the body that was not affected by LBP. *p* < .001				Participants with LBP had a statistically significant greater number of trigger points in the gluteus medius muscle compared to those without LBP, as well as, more on the affect side compared to the unaffected side (for unilateral LBP suffers).
Iglesias-Gonzalez et al. 2013 [27]	Palpation	The gluteus medius was palpated by an experience clinician. No other details reported.	Latent TrP LBP (painful side), n (% of LBP participants): *n* = 5 (12%) nLBP, n (% of nLBP participants): *n* = 2 (5%), *p* < .001 Latent TrP LBP (less painful side), n (% of LBP participants): *n* = 7 (17%) nLBP, n (% of nLBP participants): *n* = 2 (5%) *p* < .001 Active TrP LBP (more painful side), n (% of LBP participants): *n* = 15 (35%) LBP (less painful side), n (% of LBP participants): *n* = 16 (38%) *p* > .05				Participant with LBP had a statistically significant greater number of latent trigger points in both the painful and less painful sides compared to those without LBP. The number of active trigger points on either side in those with LBP was not statistically significant.
Njoo et al. 1994 [36]	Palpation	Lying prone the gluteus medius was palpated and number of trigger graded as present or absent.	LBP, n (% of LBP participants): *n* = 21 (34%) nLBP, n (% of nLBP participants): *n* = 4 (6%), *p* < .05				Participants with LBP had a statistically significant greater number of trigger points in the gluteus medius muscle compared to those without LBP.
Aboufazeli et al. 2018 [39]	Ultrasound (GE, Model GE LOGIQ S6, MA, USA), 5.0 MHz curvilinear transducer	Side-lying at rest and during resisted hip abduction (0.5Kg weightused for resistance). Only painful side measured in LBP group. In the control group, the thicknesses of both sides were measured and then averaged. Thickness was measured as the distance between the superior and inferior hyperechoic muscle fascias, at the middle of each image.	Cross-sectional area and muscle thickness				Participants with LBP demonstrated a statistically significant smaller change in gluteus medius muscle thickness, from rest to during resisted hip abduction, compared to those without LBP.
			Resting thickness LBP: 16.75 mm (0.33) nLBP: 22.00 mm (0.11) Contracted thickness LBP: 26.15 mm (0.90) nLBP: 33.90 mm (0.10) Thickness change LBP: 9.40 mm nLBP: 11.90 mm, *p* = .025* *Only reported for change between groups				
Mendis et al. 2016 [37]	1.5 T Siemens Magnetom SonataMR	Lying supine on the imaging table with knees and hips supported in neutral position.	LBP: 35.8 cm2 (±1.5) nLBP: 37.3 cm2 (±0.9), Specific statistical values not reported				No statistically significant differences between the thickness of the gluteus medius muscle between those with and without LBP.
Skorupska et al. 2016 [32]	1.5 Tesla Signa HDe system (GE)	Lying supine	No specific values for the gluteus medius muscle were reported (mm^3^).				No statistically significant differences between each side. Note: No comparisons made between groups.

*LBP* low back pain, *nLBP* no low back pain, *SD* standard deviation, *EMG* electromyography, *Mins*, *RMS* root mean squared, *MVIC* maximum voluntary isometric contraction, *MED* median, *Q1-Q3* interquartile range, *COV* coefficient of variation, *MDF* median frequency, *iEMG* integrated electromyography, *REO and LEO* right and left external oblique, *s* second, *RGMd and LGMd* right and left gluteus medius, *RIO and LIO* right and left internal oblique, *RES and LES* right and left erector spinae, *TrP* Trigger points, *n* number

#### Fatigability (EMG)

Three studies [24, 31, 34] measured the fatigability of the gluteus medius muscle with EMG (Table 2). Embaby et al. [24] found that those with LBP demonstrated statistically significant greater gluteus medius muscle fatigability after 30 min of standing compared to those without LBP, although this finding was only on the right side. Of the other two studies, one measured gluteus medius fatigability in a static weight bearing position [31], and the other in a non-weight bearing position [34], with both finding no statistically significant differences in the rate of gluteus medius muscle fatigability between those with and without LBP.

#### Time to onset (EMG)

Four studies [12, 26, 29, 30] measured the time it took for the gluteus medius muscle to activate with EMG (Table 2). Of these studies, two measured time to activation in a static single leg weight bearing position, with one requiring participants to abduct their ipsilateral hip [29], and the other study requiring participants to flex the contralateral hip [26]. Both studies found no statistically significant differences between those with and without LBP. The remaining two studies measured the time it took the gluteus medius muscle to activate during the non-weight bearing active hip abduction test [12, 30]. Nelson-Wong et al. [12] found that participants with LBP demonstrated statistically significant earlier activation of the gluteus medius, compared to some other trunk muscles (Table 2). This was in contrast to Rabel et al. [30] who found no statistically significant differences in time to activation of the gluteus medius muscle between those with and without LBP.

#### Time to peak (EMG)

One study [18] measured time to peak gluteus medius muscle activity during a static non-weight bearing kneeling task (Table 2). Participants with LBP took a statistically significant longer amount of time to reach peak activation compared to those without LBP.

### Strength

Eight studies [3–5, 16, 23, 25, 29, 33] measured the strength of the gluteus medius muscle, with two of these studies [5, 16] measuring strength in more than one way (Table 2). Of these studies, seven measured gluteus medius muscle strength in a non-weight bearing side-lying hip abduction test with participants instructed to perform maximal effort against assessor [3–5, 16, 29] or machine applied resistance [25, 33]. The majority of the studies demonstrated a statistically significant reduction in gluteus medius muscle strength in those with LBP compared to those without LBP [3–5, 16, 25, 29], with the remaining study finding no difference [33].

Two studies [16, 23] measured gluteus medius muscle strength in a static weight bearing position (Table 2). One study measured concentric strength of the gluteus medius muscle in a standing position with a dynamometer and found no difference in strength between those with and without LBP [23]. Cooper et al. [16] measured gluteus medius strength statically using the static Trendelenburg test and found that those with LBP demonstrated a positive sign more often that those without LBP (*p* < .001), indicating reduced gluteus medius muscle strength.

One study [5] measured gluteus medius muscle strength during gait using the Trendelenburg sign and found no statically significant differences between those with and without LBP.

### Trigger points in the gluteus medius muscle

Four studies [16, 27, 35, 36] investigated gluteus medius trigger points (Table 2). Of these studies, three [16, 27, 36] used manual palpation and found that those with LBP had statistically significant greater number of trigger points in the gluteus medius compared to those without LBP. The remaining study used an algometry device to measure pressure pain thresholds as a representation of areas of tenderness in the gluteus medius [35]. They found that the threshold of pressure tolerance was lower in the LBP participants compared to those without LBP (*p <* .001).

### Cross sectional area and muscle thickness

One study [39] used ultrasound to investigate the change in thickness of the gluteus medius muscle between a resting state and during resisted hip abduction (Table 2). The authors found that those with LBP demonstrated a statistically significant smaller change in muscle thickness, from rest to during resisted hip abduction, compared to those without LBP (*p* = .025).

The other two studies investigated the cross sectional muscle area of the gluteus medius muscle using magnetic resonance imaging [32, 37] (Table 2). Both studies compared side to side differences within individual participants, due to participants having unilateral LBP [32], or LBP and no LBP participants being grouped together [37], with no significant differences found.

## Discussion

This systematic review included 24 case-control studies investigating gluteus medius function in people with and without LBP. The findings for gluteus medius muscle activity [17, 18, 26, 28, 29, 31, 34, 38], fatigability [24, 31, 34], time to onset [12, 26, 29, 30], and time to peak activation [18] were mixed. Five of the eight studies measuring gluteus medius muscle strength demonstrated it to be significantly lower in those with LBP compared to those without LBP [3, 4, 16, 25, 29]. However, two of the eight studies found no difference [23, 33], and the final study had mixed findings of significantly less gluteus medius muscle strength during side-lying hip abduction in those with LBP compared to those without LBP, but no differences in strength of this muscle between these groups during the Trendelenburg test [5]. Additionally, four studies investigating the presence of trigger points [16, 27, 36] or areas of tenderness associated with trigger points [35], consistently showed that people with LBP are more likely to have higher numbers of trigger points and greater levels of tenderness in the gluteus medius muscle compared to those without LBP. The two studies that measured gluteus medius cross-sectional area found no differences [32, 37], however, another study that measured gluteus medius muscle thickness using ultrasound found that those with LBP had a significantly smaller increase in gluteus medius thickness during side-lying hip abduction [39]. Due to differences in measurement techniques (Table 2), and the type and duration of LBP (Table 1), combining studies in a meta-analysis was not possible.

The majority (9 out of 11) of studies using EMG to assess gluteus medius muscle function did so in either non-weight bearing [12, 30, 34] or static weight bearing tasks [18, 24, 26, 28, 29, 31]. Generally, only a small range of EMG variables were reported within individual studies and variables were not consistent across multiple studies. Further investigation of possible differences in dynamic gluteus medius function in those with and without LBP, and whether dynamic function is predictive of LBP development, is required to help improve our understanding of the role of this muscle in the presence and development of LBP. Inclusion of other EMG outcome variables, such as mean amplitude, minimum level of activity, or change from minimum to maximum amplitude may provide additional insight into how this muscle functions.

The reduction in gluteus medius strength reported by the majority of studies is consistent with previously reported theoretical links between biomechanical dysfunction of the lumbopelvic-hip complex and lower limb, and the development of LBP [40]. During normal gait, the gluteus medius is responsible for producing and controlling transverse plane rotation and frontal plane position of the hip joint [41]. It is proposed that weakness of the gluteus medius results in several biomechanical changes that alter the position and stability of the pelvis and may subsequently contribute to LBP [40]. In the frontal plane, gluteus medius abduction weakness, which can be seen clinically as a positive Trendelenburg sign [42], is implicated in the development of a Trendelenburg gait pattern, with the pelvis dropping to the unsupported side during single leg weight bearing in the stance phase of gait [43]. This is suggested to cause uneven distribution of pressure on intervertebral discs and subsequent loading in the lumbar joints and so contribute to the development of LBP [10, 11]. Similarly, reduced transverse plane control of the hip due to gluteus medius weakness is suggested to increase femoral adduction, internal femoral rotation and knee valgus [44, 45], causing anterior rotation of the ipsilateral pelvis, and altered lumbar spine loading, increasing the risk of LBP [46].

The consistent finding of increased numbers of active gluteus medius trigger points, as well as latent trigger points, in those with LBP [16, 27, 36] may, in part, contribute to the gluteus medius dysfunction seen in this population. Recent evidence suggests that normal patterns of motor recruitment and movement efficiency can be affected by latent trigger points [47]. Additionally, there was a positive association between the mean number of active trigger points and the mean intensity of pain episodes. This suggests that the more trigger points that are present, the greater the severity of pain and likelihood of disruption to muscle activity patterns [27].

The secondary aim of this review was to investigate differences in gluteus medius function between types and durations of LBP. However, this was hampered by inconsistent definitions of LBP and the lack of detail of LBP type and duration reported in studies. Further differences between studies, such as the method for diagnosing LBP, the tool used to assess the severity of LBP, assessment techniques, and whether or not LBP participants had pain present at the time of assessment (Table 1) are additional areas that future research should attempt to standardise so that studies can be pooled in statistical analyses [48].

### Limitations

This review was designed to be robust and comprehensive however it is possible that not all studies were identified. The likelihood of this occurring was reduced by a robust search strategy and independent title and abstract screening by two researchers. The generalisability of the review’s findings also needs to be considered. Only studies that measured gluteus medius function in participants over the age of 18 were included. This coupled with the small number of studies per measurement outcome, differences in study methodology and population, and the unclear or inconsistent definitions of the type and duration of LBP has precluded more sophisticated methods of analysis. These differences may, in part, explain some of the insignificant findings between cases and controls within studies and could also have diluted the findings of this review, perhaps explaining why our findings are unclear for some outcome measures. In addition, differing reliability of measures used may have affected the outcomes of the included studies. Although it was not the purpose of this review to determine measurement reliability, only ten of the included studies reported measurement reliability, with large variability between studies for the same measurement outcome [3, 16, 25, 26, 30, 32, 35–37, 39]. Poor reliability of any measurement can account for insignificant findings where differences between cases and controls are small. This may be relevant to the results of studies included in this review and we suggest a comprehensive investigation of the existing reliability in this area be undertaken. The findings of this systematic review should be interpreted with caution and in context of the limitations of the review itself and those of the individual studies. Nevertheless, this systematic review provides a summary of the available literature which can be used to inform both clinical practice and future research.

## Conclusion

In summary, we found that in those with LBP the gluteus medius muscle had reduced strength and more trigger points compared to those without LBP. Findings for the level of muscle activity, fatigability, time to onset, time to peak amplitude, cross sectional area, and muscle thickness were mixed. When interpreting these findings in context of the management of LBP patients, significant caution is recommended because the aim of this review was not to investigate intervention effectiveness. However, strengthening the gluteus medius muscle and eliminating trigger points may form an important part of the multidisciplinary management of LBP patients, although further research is needed before this can be confidently recommended. To help reduce inconsistencies in future research, the authors recommended following the standardised eligibility criteria outlined by Amundsen et al. [48]. Additionally, future research should aim to prospectively assess gluteus medius muscle function, with static and dynamic tasks across a range of outcome measures, and in those with and without LBP.

## Supplementary information

**Additional file 1.** Key word search.

**Additional file 2.** Quality appraisal.

**Additional file 3.** Table of excluded full text articles.

## Data Availability

All of the data for this study are contained in the manuscript, the additional files, or the individual studies included in this systematic review.

## Abbreviations

EMG: Electromyography
LBP: Low back pain
