# Supplementary material for: Gluteus medius muscle function in people with and without low back pain: a systematic review
Source: BMC Musculoskelet Disord. 2019 Oct 22;20:463. doi: 10.1186/s12891-019-2833-4 (PMC6805550; doi:10.1186/s12891-019-2833-4)
Supplement: Supplementary file 1 — Additional file 1. Key word search. [file 12891_2019_2833_MOESM1_ESM.docx]

| **Additional file 1**: Key words and truncations for database search. | |
| --- | --- |
| Hip muscle* | **OR** |
| Lumbopelvic muscle* |  |
| Pelvic muscle* |  |
| Gluteus medius |  |
| Hip abductor* |  |
| Hip external rotator* |  |
| Hip internal rotator* |  |
|  | **AND** |
| Low* back trouble | **OR** |
| LBT |  |
| LBP |  |
| Back pain |  |
| Chronic low* back pain |  |
| Acute low* back pain |  |
| Subacute low* back pain |  |
| Nonspecific low* back pain |  |
| Specific low* back pain |  |
