# Supplementary material for: Gluteus medius muscle function in people with and without low back pain: a systematic review
Source: BMC Musculoskelet Disord. 2019 Oct 22;20:463. doi: 10.1186/s12891-019-2833-4 (PMC6805550; doi:10.1186/s12891-019-2833-4)
Supplement: Supplementary file 2 — Additional file 2. Quality appraisal. [file 12891_2019_2833_MOESM2_ESM.docx]

| \| **Additional file 2: Quality appraisal (part 1)** \| \| \| \| \| --- \| --- \| --- \| --- \| \|  \| Aboufazeli et al. 2018[39] \| Arab et al. 2010[4] \| Cai et al. 2015[23] \| \| Cooper et al. 2016[16] \| Embaby et al. 2013[24] \| Farahpour et al. 2018[17] \| Farasyn et al. 2005[35] \| Hides et al. 2016[25] \| Hungerford et al. 2003[26] \| Iglesias-Gonzalez et al. 2013[27] \| Kendall et al. 2010[5] \| Larsen et al. 2018[38] \| \| 1. Did the study address a clearly focused issue? \| Yes \| Yes \| Yes \| \| Yes \| Yes \| Yes \| Yes \| Yes \| Yes \| Yes \| Yes \| Yes \| \| 2. Did the authors use an appropriate method to answer their questions? \| Yes \| Yes \| Yes \| \| Yes \| Yes \| Yes \| Yes \| Yes \| Yes \| Yes \| Yes \| Yes \| \| 3. Were the cases recruited in an acceptable way? \| Yes \| Yes \| Yes \| \| Yes \| Yes \| Yes \| Yes \| Yes \| Yes \| Yes \| Yes \| Yes \| \| 4. Were the controls selected in an acceptable way? \| Yes \| Yes \| Yes \| \| Yes \| Can’t tell \| Yes \| Can’t Tell \| Yes \| Yes \| Yes \| Can’t tell \| Yes \| \| 5. Was the exposure accurately measured to minimise bias? \| Yes \| No \| Yes \| \| Can’t tell \| Yes \| Yes \| Yes \| Yes \| Yes \| Yes \| Yes \| Yes \| \| 6 (a). What confounding factors have the authors accounted for? \| Age, height, weight, & BMI \| BMI, ITB tightness, & LBP \| Sex & age \| \| Sex & BMI \| Age, weight, & height \| Age, BMI, & foot posture \| BMI & ODI \| Muscle size & strength \| Age, sex, & height \| Age & sex \| Age & sex \| Age, BMI \| \| 6 (b). Have the authors taken account of the potential confounding factors in the design and/or in their analysis? \| Yes \| Yes \| Yes \| \| Yes \| Can’t tell \| Yes \| Yes \| Yes \| Yes \| Yes \| Yes \| Yes \| \| 7. What are the results of the study? \| Change in gluteus medius thickness during hip abduction smaller in LBP group vs control group \| Hip abductor weaker in LBP group vs control group \| No difference in hip abductor torque between groups \| \| Reduced hip abductor strength in LBP vs control group \| Reduced EMG activity gluteus medius in LBP vs control group \| Increased EMG activity in LBP vs control group \| Pressure pain threshold reduced in LBP vs controls group \| Inverse hip abductor strength between stance & kicking leg in LBP vs Control \| No difference in EMG activity between LBP and control groups \| Latent TrP more common in LBP vs controls group \| Reduced hip abduction strength in LBP vs controls control \| No difference in EMG activity in LBP and control groups \| \| 8. How precise are the results?  How precise is the estimate of risk? \| Can’t tell, *P*=0.025 \| Can’t tell, *P*<0.001 \| Can’t tell, *P*=0.596 \| \| Can’t tell, *P*<0.001 \| Can’t tell, *p*<0.05 \| Can’t tell, *p*<0.05 \| Can’t tell, *P* <0.001 \| Can’t tell, *P* =0.04 \| Can’t tell, *P*>0.05 \| Can’t tell, *p*<0.001 \| Can’t tell, *p*>0.05 \| Can’t tell, *p*>0.05 \| \| 9. Do you believe the results? \| Yes \| Yes \| Can’t tell \| \| Yes \| Yes \| Yes \| Yes \| No \| Yes \| Yes \| Yes \| Yes \| \| 10. Can the results be applied to the local population? \| Yes \| Yes \| Yes \| \| Yes \| Yes \| Yes \| Yes \| No \| Yes \| Yes \| Yes \| Yes \| \| 11. Do the results of this study fit with other available evidence? \| Can’t tell \| Yes \| Can’t tell \| \| Yes \| Can’t tell \| Can’t tell \| Can’t tell \| Yes \| Yes \| Yes \| Yes \| Can’t tell \|   **Additional file 2 (continued): Quality appraisal (part 2)** | | | | | | | | | | | | |
| --- | --- | --- | --- | --- | --- | --- | --- | --- | --- | --- | --- | --- | --- | --- | --- | --- | --- | --- | --- | --- | --- | --- | --- | --- | --- | --- | --- | --- | --- | --- | --- | --- | --- | --- | --- | --- | --- | --- | --- | --- | --- | --- | --- | --- | --- | --- | --- | --- | --- | --- | --- | --- | --- | --- | --- | --- | --- | --- | --- | --- | --- | --- | --- | --- | --- | --- | --- | --- | --- | --- | --- | --- | --- | --- | --- | --- | --- | --- | --- | --- | --- | --- | --- | --- | --- | --- | --- | --- | --- | --- | --- | --- | --- | --- | --- | --- | --- | --- | --- | --- | --- | --- | --- | --- | --- | --- | --- | --- | --- | --- | --- | --- | --- | --- | --- | --- | --- | --- | --- | --- | --- | --- | --- | --- | --- | --- | --- | --- | --- | --- | --- | --- | --- | --- | --- | --- | --- | --- | --- | --- | --- | --- | --- | --- | --- | --- | --- | --- | --- | --- | --- | --- | --- | --- | --- | --- | --- | --- | --- | --- | --- | --- | --- | --- | --- | --- | --- | --- | --- | --- | --- | --- | --- | --- | --- | --- | --- | --- | --- | --- | --- | --- | --- | --- | --- | --- | --- | --- | --- | --- | --- | --- | --- | --- | --- | --- | --- | --- |
|  | Mendis et al. 2016[37] | Nelson-Wong et al. 2013[6] | Njoo et al. 1994[36] | Notzel et al. 2011[28] | Nourbakhsh et al. 2002[3] | Penney et al. 2014[29] | Rabel et al. 2013[30] | Ringheim et al. 2015[31] | Santos et al. 2013[18] | Skorupska et al. 2016[32] | Sutherlin et al. 2015a[33] | Sutherlin et al. 2015b[34] |
| 1. Did the study address a clearly focused issue? | Yes | Yes | Yes | Yes | Yes | Yes | Yes | Yes | Yes | Yes | Yes | Yes |
| 2. Did the authors use an appropriate method to answer their questions? | Yes | Yes | Yes | Yes | Yes | Yes | Yes | Yes | Yes | Yes | Yes | Yes |
| 3. Were the cases recruited in an acceptable way? | Yes | Yes | Yes | Can’t Tell | Yes | Yes | Yes | Yes | Yes | Yes | Yes | Yes |
| 4. Were the controls selected in an acceptable way? | Can’t Tell | Yes | Can’t tell | Can’t Tell | Yes | Yes | Can’t Tell | Yes | Yes | Can’t Tell | Can’t Tell | Can’t Tell |
| 5. Was the exposure accurately measured to minimise bias? | Yes | Can’t Tell | No | Can’t Tell | Can’t Tell | Yes | Can’t Tell | Yes | Can’t Tell | Yes | Yes | Yes |
| 6 (a). What confounding factors have the authors accounted for? | Age, weight, height, & sex | Age, sex, & BMI | Can’t tell | Can’t tell | Age | Age, weight, & height | Age | Can’t tell | Age, weight, & BMI | Can’t tell | Can’t tell | Can’t tell |
| 6 (b). Have the authors taken account of the potential confounding factors in the design and/or in their analysis? | Yes | Yes | Yes | Can’t tell | Yes | Yes | Yes | Can’t Tell | Can’t Tell | Yes | Yes | Yes |
| 7. What are the results of the study? | No comparisons made between groups for gluteus medius | Altered gluteus medius activation for LBP vs control group | Increased TrP in LBP vs control group | Reduced gluteus medius EMG activity in LBP vs controls group | Reduced gluteus medius strength and more EMG activity in LBP vs control group | Reduced hip abduction strength in LBP vs controls group | No difference in gluteus medius EMG activity between LBP and control group | No difference in EMG activity in LBP vs control group | Reduced EMG activity of gluteus medius in LBP vs controls group | No difference in muscle volume for gluteus medius in LBP vs control group | No difference in hip abduction torque in LBP vs controls group | No difference in EMG activity of gluteus medius in LBP vs controls group |
| 8. How precise are the results?  How precise is the estimate of risk? | Can’t tell, *P* <0.02 | Can’t tell *p*<0.05 | Can’t tell *p*<0.05 | Can’t tell *p*<0.05 | Can’t tell *p*<0.05 | Can’t tell *p*<0.05 | Can’t tell *p*=0.115 | Can’t tell *p*>0.05 | Can’t tell *p*<0.007 | Can’t tell *p*>0.05 | Can’t tell *p*>0.05 | Can’t tell *p*>0.05 |
| 9. Do you believe the results? | Yes | Yes | No | Can’t tell | Can’t tell | Yes | Can’t tell | Can’t tell | Yes | Yes | Yes | Yes |
| 10. Can the results be applied to the local population? | No | Yes | Yes | Can’t tell | Yes | Yes | Yes | Yes | Yes | Yes | Yes | No |
| 11. Do the results of this study fit with other available evidence? | Yes | Yes | Yes | Can’t tell | Yes | Can’t tell | Can’t tell | No | Yes | Yes | No | Yes |
