# Supplementary material for: Gluteus medius muscle function in people with and without low back pain: a systematic review
Source: BMC Musculoskelet Disord. 2019 Oct 22;20:463. doi: 10.1186/s12891-019-2833-4 (PMC6805550; doi:10.1186/s12891-019-2833-4)
Supplement: Supplementary file 3 — Additional file 3. Table of excluded full text articles. [file 12891_2019_2833_MOESM3_ESM.docx]

|  | Author | Title | Grounds for Exclusion |
| --- | --- | --- | --- |
|  | Aghazadeh, J., et al. (2015). | "Anti-fatigue mats, low back pain, and electromyography: An interventional study." | Not a Case Control Study |
|  | Aly, S. M., et al. (2017). | "Effect of Six Weeks of Core Stability Exercises on Trunk and Hip Muscles’ Strength in College Students." | Not a Case Control Study |
|  | Amabile, C., et al. (2017). | "Estimation of spinopelvic muscles' volumes in young asymptomatic subjects: a quantitative analysis." | Not a Case Control Study |
|  | Amir Massoud, A., et al. (2017). | "Lumbar lordosis in prone position and prone hip extension test: comparison between subjects with and without low back pain." | Gluteus medius muscle not measured |
|  | Anders, C., et al. (2009). | "Healthy humans use sex-specific co-ordination patterns of trunk muscles during gait." | Not a Case control study |
|  | Antle, D. M. and J. N. Cote (2013). | "Relationships between lower limb and trunk discomfort and vascular, muscular and kinetic outcomes during stationary standing work” | Not a Case control study |
|  | Arab, A. M., et al. (2011). | "Altered muscular activation during prone hip extension in women with and without low back pain." | Gluteus medius muscle not measured |
|  | Bach et al. 1985 | A comparison of muscular tightness in runners and nonrunners and the relation of muscular tightness to low back pain in runners. | No separate data for those with and without LBP |
|  | Barbosa, A. C., et al. (2016). | "Increased Activation Amplitude Levels of Gluteus Medius in Women During Isometric and Dynamic Conditions Following a 4-week Protocol of Low-load Eccentric Exercises." | Not a Case Control Study |
|  | Beazell, J. P. T. D. P. T. O. C. S. F. A. T. C., et al. (2011). | "Exercise-Related Postural Control Deficits in Individuals with Recurrent Low Back Pain." | Gluteus medius muscle not measured |
|  | Bhura, P. A. and C. A. Bhagat (2014). | "A Study on Iliotibial Band Tightness in Postural Low Back Pain." | Gluteus medius muscle not measured |
|  | Broadbent S, Coutts R, Coetzee S | Targeted exercise interventions in improving injuries, flexibility and strength in female dragon boat paddlers. | Not a Case Control Study |
|  | Bullock-Saxton, J. E., et al. (1993). | "Reflex activation of gluteal muscles in walking. An approach to restoration of muscle function for patients with low-back pain." | Not a Case Control Study |
|  | Bussey, M., et al. (2016) | “Gluteus medius coactivation response in field hockey players with and without low back pain” | Not a Case Control Study |
|  | Cambridge, E. D. J., et al. (2012). | "Progressive hip rehabilitation: The effects of resistance band placement on gluteal activation during two common exercises." | Not A Case Control Study |
|  | Chacon, M.E. (1990) | “The relationship of trunk and hip muscle strength to low back pain” | Unable to Retrieve Full Text |
|  | Ciesielska, J. et al. (2015) | “Hip strategy alterations in patients with history of low disc herniation and non-specific low back pain measured by surface electromyography and balance platform” | Gluteus medius muscle not measured |
|  | de Aquino, C. F., et al. (2010). | "Analysis of the relationship between low back pain and muscle strength imbalance in ballet dancers." | Gluteus medius muscle not measured |
|  | Dulger, E. et al. (2018) | “The effect of stabilization exercises on diaphragm muscle thickness and movement in women with low back pain” | Gluteus medius muscle not measured |
|  | Dwyer, M. K. P. A. T. C., et al. (2010). | "Comparison of Lower Extremity Kinematics and Hip Muscle Activation During Rehabilitation Tasks Between Sexes." | Not a Case Control Study |
|  | Elerian, A. E., et al. (2016). | “Comparison between the different hip rotation positions on hip abduction exercise.” | Not a Case Control Study |
|  | Eun Hyuk, K., et al. (2015). | "Effect of Hip Abduction Exercise with Manual Pelvic Fixation on Recruitment of Deep Trunk Muscles." | Not a Case Control Study |
|  | Fasuyi, F. O., et al. (2017). | “Hamstring muscle length and pelvic tilt range among individuals with and without low back pain.” | Gluteus medius muscle not measured |
|  | Flack, N. A., et al. (2012). | "Hip abductor muscle volume in women with lateral hip pain: a case-controlled study." | Does not include participants with and without LBP |
|  | Freddolini, M., et al. (2014). | "The role of trunk muscles in sitting balance control in people with low back pain." | Gluteus medius muscle not measured |
|  | Fujitani, R., et al. (2017). | "Effect of standing postural deviations on trunk and hip muscle activity.” | Not a Case Control Study |
|  | Gasibat, Q., et al. (2017) | “Effect of the stabilization training programme in the improvement of trunk and hip muscles activations among healthy females subject: An implication for the rehabilitation experts.” | Not a Case Control Study |
|  | Güell, J. J., et al. (2011). | "Lumbalgia crónica inespecífica. Tests físicos para detectarla. Prueba piloto/Chronic Nonspecific Low Back Pain: Physical Tests to Detect It. Pilot Study." | Gluteus medius muscle not measured |
|  | Guimarães, C. Q., et al. (2010). | "Electromyographic activity during active prone hip extension did not discriminate individuals with and without low back pain." | Gluteus medius muscle not measured |
|  | Harithasan, D., et al. (2017). | "Effects of back belt on vertical load transfer among adults with non-specific low back pain during asymmetrical manual load carrying." | Gluteus medius muscle not measured |
|  | Hooper, T. L., et al. (2016). | "Dynamic balance as measured by the Y-Balance Test is reduced in individuals with low back pain: A cross-sectional comparative study." | Gluteus medius muscle not measured |
|  | Hu, H., et al. (2010). | "Muscle activity during the active straight leg raise (ASLR), and the effects of a pelvic belt on the ASLR and on treadmill walking." | Gluteus medius muscle not measured |
|  | Kahlaee, A. H., et al. (2017). | "Effect of the Abdominal Hollowing and Bracing Maneuvers on Activity Pattern of the Lumbopelvic Muscles During Prone Hip Extension in Subjects With or Without Chronic Low Back Pain: A Preliminary Study." | Gluteus medius muscle not measured |
|  | Kamaz, M., et al. (2007). | "CT measurement of trunk muscle areas in patients with chronic low back pain." | Gluteus medius muscle not measured |
|  | Kim, J.-W., et al. (2014). | "Patients with low back pain demonstrate increased activity of the posterior oblique sling muscle during prone hip extension." | Gluteus medius muscle not measured |
|  | Kim, J.-W., et al. (2014). | "Effects of external pelvic compression on trunk and hip muscle EMG activity during prone hip extension in females with chronic low back pain." | Gluteus medius muscle not measured |
|  | Kiran, R., et al. (2017). | “Thoracic mobilisation and periscapular soft tissue manipulations in the management of chronic Prolapsed Intervertebral Disc (PIVD) - An innovative manual therapy approach.” | Gluteus medius muscle not measured |
|  | Larivière, C., et al. (2010). | "Specificity of a back muscle exercise machine in healthy and low back pain subjects." | Gluteus medius muscle not measured |
|  | Lee, S. and D. Park (2013). | "The effects of knee joint and hip abduction angles on the activation of cervical and abdominal muscles during bridging exercises." | Not a Case Control Study |
|  | Lee, N. G., et al. (2015). | "Unipedal postural stability in nonathletes with core instability after intensive abdominal drawing-in maneuver." | Not a Case Control Study |
|  | Lemaire, A., et al. (2013). | "Relationships between hip muscles and trunk flexor and extensor muscles in chronic low back pain patients: a preliminary study." | Not a Case Control Study |
|  | Mangum LC, Murray KP, Saliba SA | Mechanical and Electrical Evaluation of Muscle Activity During Exercise in Individuals With and Without Low Back Pain | Unable to retrieve Full Text |
|  | Marshall, P. W. M., et al. (2011). | "Gluteus medius strength, endurance, and co-activation in the development of low back pain during prolonged standing." | Does not include participants with and without LBP |
|  | Mens, J. M. A., et al. (2002). | "Responsiveness of outcome measurements in rehabilitation of patients with posterior pelvic pain since pregnancy." | Not a Case Control Study |
|  | Morini, S., et al. (2008). | "Functional anatomy of trunk flexion-extension in isokinetic exercise: muscle activity in standing and seated positions." | Not a Case Control Study |
|  | Nadler, S., et al. (2001). | "Relationship between hip muscle imbalance and occurrence of low back pain in collegiate athletes: a prospective study." | No separate data for those with and without LBP |
|  | Nadler, S. F., et al. (2000). | "The relationship between lower extremity injury, low back pain, and hip muscle strength in male and female collegiate athletes." | No separate data for those with and without LBP |
|  | Nelson-Wong, E., et al. (2008). | "Gluteus medius muscle activation patterns as a predictor of low back pain during standing." | Not a Case Control Study |
|  | Nelson-Wong, E. and J. P. Callaghan (2010). | "Changes in muscle activation patterns and subjective low back pain ratings during prolonged standing in response to an exercise intervention." | Not a Case Control Study |
|  | Nelson-Wong, E. and J. P. Callaghan (2010). | "Is muscle co-activation a predisposing factor for low back pain development during standing? A multifactorial approach for early identification of at-risk individuals." | Not a Case Control Study |
|  | Nelson-Wong, E. and J. P. Callaghan (2010). | "Repeatability of Clinical, Biomechanical, and Motor Control Profiles in People with and without Standing-Induced Low Back Pain." | Not a Case Control Study |
|  | Nelson-Wong, E. and J. P. Callaghan (2010). | "The impact of a sloped surface on low back pain during prolonged standing work: A biomechanical analysis." | Not a Case Control Study |
|  | Norén, L., et al. (2002). | "Lumbar back and posterior pelvic pain during pregnancy: a 3-year follow-up." | Not a Case Control Study |
|  | Nourbakhsh, M. R., et al. (2006). | "The relationship between pelvic cross syndrome and chronic low back pain." | Gluteus medius muscle not measured |
|  | Paatelma, M., et al. (2009). | "Clinical perspective: how do clinical test results differentiate chronic and subacute low back pain patients from 'non-patients'?" | Gluteus medius muscle not measured |
|  | Paquet, N., et al. (1994). | "Hip-spine movement interaction and muscle activation patterns during sagittal trunk movements in low back pain patients." | Gluteus medius muscle not measured |
|  | Park, K. M., et al. (2010). | "Effects of the pelvic compression belt on gluteus medius, quadratus lumborum, and lumbar multifidus activities during side-lying hip abduction." | Not a Case Control Study |
|  | Petrofsky, J. S., et al. (2008). | "Improving the outcomes after back injury by a core muscle strengthening program." | Gluteus medius muscle not measured |
|  | Pirouzi, S., et al. (2006). | "Low back pain patients demonstrate increased hip extensor muscle activity during standardized submaximal rotation efforts." | Gluteus medius muscle not measured |
|  | Poulsen, K. M. (2016). | "Biodynamic parameters during a step down task in subjects with chronic or recurrent low back pain classified with lumbar instability." | Not a published peer reviewed article or conference abstract |
|  | Roussel, N., et al. (2013). | "Motor Control and Low Back Pain in Dancers." | Participants <18 years included |
|  | Schellenberg, F., et al. (2017). | "Loading conditions in the spine, hip and knee during different executions of back extension exercises." | Not a Case Control Study |
|  | Shum, G. L., et al. (2007). | "Three-dimensional kinetics of the lumbar spine and hips in low back pain patients during sit-to-stand and stand-to-sit." | No separate data for those with and wihout low back pain |
|  | Stewart, D. M. and D. E. Gregory (2016). | "The use of intermittent trunk flexion to alleviate low back pain during prolonged standing." | Not a Case Control Study |
|  | Suehiro, T., et al. (2015). | "Individuals with chronic low back pain demonstrate delayed onset of the back muscle activity during prone hip extension." | Gluteus medius muscle not measured |
|  | Ui-Cheol, J., et al. (2015). | "The effects of gluteus muscle strengthening exercise and lumbar stabilization exercise on lumbar muscle strength and balance in chronic low back pain patients." | Not a Case Control Study |
|  | Van Meeteren J, Mens JMA, Stam HJ: | “Reliability of strength measurement of the hip with a hand-held dynamometer in healthy women.” | Not a Case Control Study |
|  | Viggiani, D. and Callaghan, J. P. (2016). | "A hip abduction exercise prior to prolonged standing increased movement while reducing cocontraction and low back pain perception in those initially reporting low back pain." | Not a Case Control Study |
|  | Viggiani, D. and Callaghan, J. P. (2018). | “Hip Abductor Fatigability and Recovery are Related to the Development of Low Back Pain During Prolonged Standing.” | Not a Case Control Study |
|  | Youdas, J. W., et al. (2014). | "Surface electromyographic analysis of core trunk and hip muscles during selected rehabilitation exercises in the side-bridge to neutral spine position." | Not a Case Control Study |
